# Gluteal hidradenitis suppurativa presenting pemphigus-like findings: case report

**DOI:** 10.1186/s12895-019-0091-7

**Published:** 2019-07-23

**Authors:** Yuichi Kurihara, Masutaka Furue

**Affiliations:** 1 Department of Dermatology, Steel Memorial Yawata Hospital, 1-1-1 Harunomachi, Yahatahigashi-ku, Kitakyushu, 805-8508 Japan; 2 Department of Dermatology, Graduate School of Medical Sciences, Kyushu University, 3-1-1 Maidashi, Higashi-ku, Fukuoka, 812-8582 Japan

**Keywords:** Hidradenitis suppurativa, Pemphigus, Acantholysis, Desmoglein autoantibodies

## Abstract

**Background:**

Hidradenitis suppurativa is one member of the follicular occlusion triad: acne conglobata, hidradenitis suppurativa, and dissecting cellulitis of the scalp. The presence of acantholysis and desmoglein autoantibodies in hidradenitis suppurativa is rare.

**Case presentation:**

We report a case of 68-year-old male with a diagnosis of gluteal hidradenitis suppurativa co-presenting pemphigus-like findings including acantholysis and positive desmoglein autoantibodies.

**Conclusion:**

To our knowledge, comorbidity of gluteal hidradenitis suppurativa and pemphigus-like findings has not been reported before. This case implies a relationship between two different conditions; the follicular occlusion triad and pemphigus, highlighting a potential induction of pemphigus-like lesion by chronic inflammatory process.

## Background

Hidradenitis suppurativa is one member of the follicular occlusion triad: acne conglobata, hidradenitis suppurativa, and dissecting cellulitis of the scalp [1]. This group of conditions presents with deep scarring folliculitis composed of multichannel draining sinus and abscesses [1]. Hidradenitis suppurativa is a progressive inflammatory disease and emerges comorbid autoimmune disease [2]. We report a rare case of gluteal hidradenitis suppurativa which also presented acantholysis and desmoglein autoantibodies.

## Case presentation

**Fig. 1:**
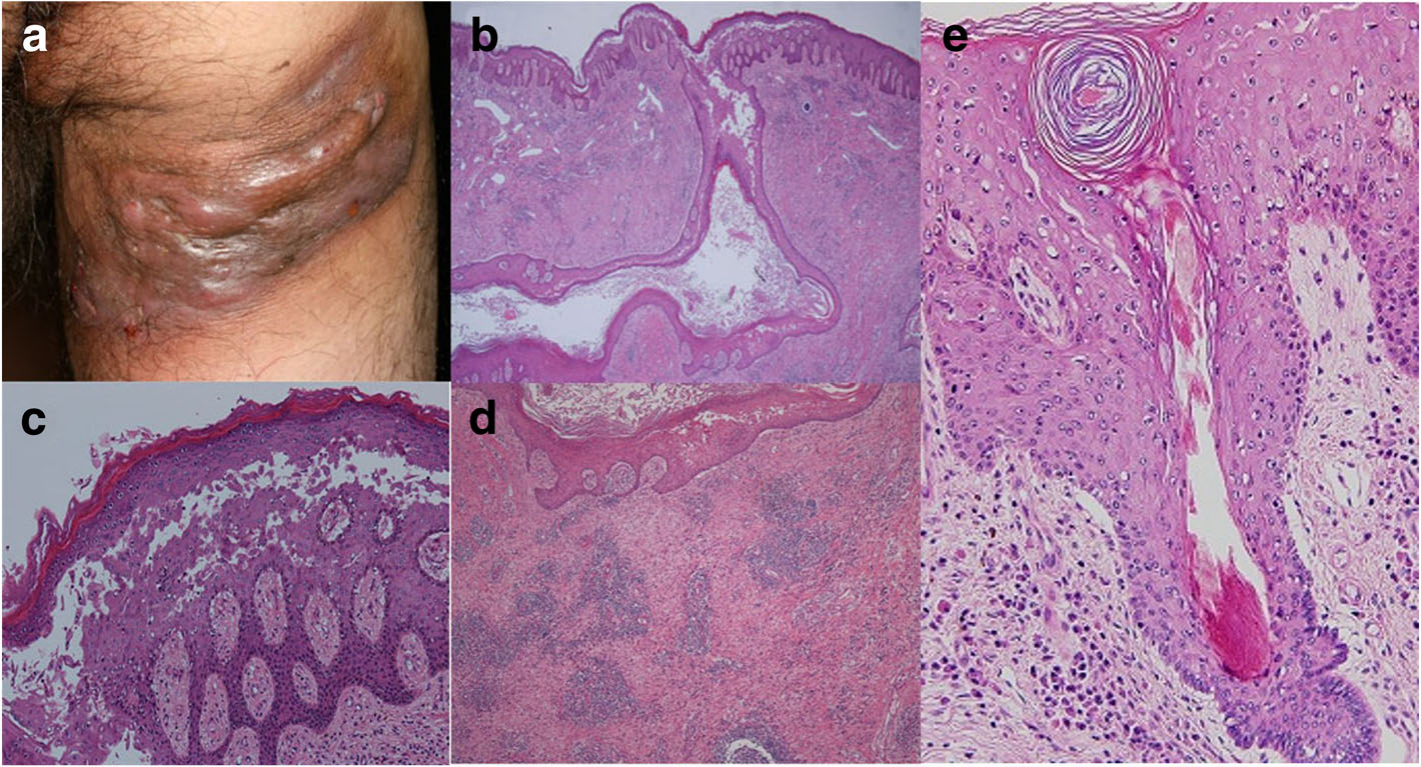
(**a**) Brown nodules, multiple fistula and scars on the right side of the thigh. (**b**) Clefts and acantholysis in the epidermis (hematoxylin and eosin, original magnification × 40). (**c**) Presence of intraepidermal acantholytic cells (original magnification × 200) (**d**) Abscesses and chronic inflammation in the upper dermis. (hematoxylin and eosin, original magnification × 100). (**e**) Involvement of hair follicle in the lesion (original magnification × 200)

A 68-year-old male presented slightly tender brown nodules and purulent discharge on the right thigh. There were no evident blisters or erosions. He had first developed these lesions three years previously and they had been gradually enlarged. Physical examination revealed brown nodules, multiple fistulae, and scars on the right side of the thigh (Fig. 1a). No other areas of the body including mucosal areas were affected. The patient did not report any gastrointestinal symptoms and had no remarkable past medical history including immunodeficiency and relevant family history. *Staphylococcus aureus* was cultured from the lesion but oral antibiotic treatment for over three months was ineffective, then surgical removal of the lesion was performed. The whole lesion had been totally excised and processed for formalin-fixation. Therefore, standard direct immunofluoresence test was not performed. Indirect immunofluorescence finding was negative. The results of serum ELISA tests for anti-desmoglein 1 and 3 (MESACUP desmoglein 1 and desmoglein 3, MBL) were positive (ELISA titers were 36 and 11, respectively) and epidermal hyperplasia, numerous intraepidermal clefts and acantholysis were demonstrated in histology (Fig. 1b, c). Furthermore, the findings of gluteal hidradenitis suppurativa, namely, multiple sinus tracts, abscesses, chronic inflammation with polymorphonuclear leukocytes and lymphocytes, and follicle-based inflammation were observed (Fig. 1d, e). Considering the age of onset, unilaterality of lesion and the site and extent of lesion, hidradenitis suppurativa was compatible rather than acne conglobata. At the time of writing, no signs of recurrence after the surgical treatment have emerged.

The differential diagnosis includes pyoderma vegetans, pemphigus vegetans, pyodermatitis-pyostomatitis and other autoimmune blistering dermatoses. In the present case, the absence of mucosal lesions, abdominal symptoms, and eosinophilia ruled out the diagnosis of pyoderma vegetans and pyodermatitis-pyostomatitis. The response to oral antibiotics was poor and ruled out infected pemphigus vegetans. We diagnosed this case as hidradenitis suppurativa with pemphigus-like findings because of the unilaterally-localized lesion, low titer of desmoglein antibodies and negative indirect immunofluorescence finding, although concomitant presence of hidradenitis suppurativa and pemphigus could not be ruled out exactly.

## Discussion and conclusion

Although the precise pathogenesis is not fully understood, a previous study described the pemphigus-pyoderma spectrum [3]. The present case also suggests a potential (at least coincidental) relationship between the follicular occlusion triad and pemphigus-like findings. Interestingly, although the blood test results were positive for IgG antibody to desmoglein 1 and 3, the lesion was confined to the thigh. A previous report indicated the association between recurrent staphylococcal infection and the development of an anti-desmoglein 3 antibody response [4, 5]. It was possible that recurrent bacterial infections may influence or aggravate the intraepidermal lesions in the present case. Finally, the present case may highlight a possibility that pemphigus-like findings can be triggered or induced by chronic inflammatory process like hidradenitis supprativa.

## Data Availability

All data is included within the published article.
